# Supplementary material for: Implementation of National Nutrition Policies and Strategies to Reduce Unhealthy Diets: An Ecological Analysis of 194 Countries from 2017 to 2021
Source: Nutrients. 2024 Mar 21;16(6):911. doi: 10.3390/nu16060911 (PMC10975650; doi:10.3390/nu16060911)
Supplement: Supplementary file 1 [file nutrients-16-00911-s001.zip › nutrients-2924460-supplementary.pdf]

Table S1. Indicators included in the WHO's NCD Progress Reports and Country Capacity Surveys

The following is a list of indicators that are included in the NCD Progress Reports		Selection in current study
1	The Member State should set time-bound national targets based on guidance from the World Health Organization.	Included
2	The Member State should have a reliable system for collecting data on the causes of death.	Included
3	The Member State should conduct a health examination survey every 5 years using the STEPwise approach to surveillance (STEPS).	Included
4	The Member State should have a national strategy/action plan that integrates the major NCDs and their shared risk factors across multiple sectors.	Included
5	The Member State should implement measures to:	
5A	Reduce tobacco's affordability.	
5B	Eliminate exposure to second-hand smoke	
5C	Use plain/standardized packaging and graphic health warnings	
5D	Ban tobacco advertising, promotion, and sponsorship	
5E	Educate the public about the harms of smoking/tobacco use and second-hand smoke through mass media campaigns	
6	The Member State should enact and enforce:	
6A	restrictions on the physical availability of alcohol	
6B	restrictions on exposure to alcohol advertising	
6C	the increase of excise taxes on alcoholic beverages	
7	The Member State should adopt national policies to:	
7A	Reduce population salt/sodium consumption	Included
7B	Limit saturated fatty acids and virtually eliminate industrially-produced trans-fatty acids in the food supply	Included
7C	Implement the WHO set of recommendations on marketing of foods and non-alcoholic beverages to children	Included
7D	Have legislation/regulations fully implementing the International Code of Marketing of Breast-milk Substitutes.	Included
8	The Member State should have up-to-date national public awareness programs and motivational communication strategies that promote physical activity. This includes running mass media campaigns that aim to encourage positive behavioral changes towards regular physical activity.	
9	The Member State should have evidence-based national guidelines/protocols/standards for the management of major NCDs through a primary care approach that are recognized or approved by government or competent authorities.	
10	The Member State should provide drug therapy and counselling for eligible persons at high risk to prevent heart attacks and strokes, with emphasis on the primary care level.	
**The following is a list of indicators that are included in the NCD National Capacity Surveys**		

**The infrastructure's capacity at the national level for NCD**		
1	The Ministry of Health has an operational unit, branch, or department responsible for NCDs.	Included
2	A national multisectoral commission, agency, or mechanism is established to address NCDs at the national level.	Included
3	A tax on sugar-sweetened beverages is implemented in the country.	Included
4	A tax on foods high in fat, sugars, or salt is implemented in the country.	Included (Data available only for 2021)
5	The availability of price subsidies for healthy foods is ensured.	Included (Data available only for 2021)
6	Tax incentives are introduced to promote physical activity.	
**Policies, strategies and action plans**		
1	The Member State has an active national policy, strategy or action plan that addresses multiple non-communicable diseases (NCDs) and their risk factors across sectors.	Present in the NCD Progress Reports
2	The Member State has established time-bound national targets aligned with WHO guidance.	Present in the NCD Progress Reports
3	The Member State has implemented measures to reduce salt consumption among its population.	Present in the NCD Progress Reports
4	The Member State has implemented policies that aim to regulate the marketing of foods to children.	Present in the NCD Progress Reports
5	The Member State has an operational policy, strategy, or action plan that addresses cardiovascular diseases.	
6	The Member State has established an operational policy, strategy, or action plan that addresses cancer.	
7	The Member State has established an operational policy, strategy, or action plan that addresses diabetes.	
8	The Member State has established an operational policy, strategy, or action plan that addresses chronic respiratory diseases.	
9	The Member State has implemented measures to reduce the harmful use of alcohol.	
10	The Member State has established an operational policy, strategy, or action plan that addresses unhealthy diets related to NCDs.	Included
11	The Member State has established an operational policy, strategy, or action plan that aims to decrease tobacco use.	
12	The Member State has implemented national policies to regulate saturated fatty acids.	Present in the NCD Progress Reports

13	The Member State has implemented national policies to eliminate trans-fatty acids.	Present in the NCD Progress Reports
14	The Member State has established an operational policy, strategy, or action plan that addresses oral health.	
15	The Member State has established an operational policy, strategy, or action plan that addresses eye health.	
16	The Member State has established an operational policy, strategy, or action plan that addresses hearing health.	
17	The Member State has established an operational policy, strategy, or action plan that aims to reduce overweight or obesity.	
18	The Member State has established an operational policy, strategy, or action plan that aims to reduce physical inactivity.	
19	The Member State has implemented a public awareness program to promote physical activity.	
20	The Member State has national guidelines for physical activity.	
21	The Member State has national guidelines for physical activity for children under five years old.	
22	The Member State has national guidelines for physical activity for children and adolescents aged 5-19.	
23	The Member State has national guidelines for physical activity for adults.	
24	The Member State has national guidelines for physical activity for older adults.	
25	The Member State has policies that encourage walking and cycling as a means to promote physical activity.	
26	The Member State has policies that promote physical activity in workplaces.	
27	The Member State has policies that promote physical activity in public open spaces.	
28	The Member State has policies that promote physical activity in community-based and sports initiatives.	
29	The Member State has policies that promote physical activity in childcare settings.	
30	The Member State has policies that promote physical activity in active ageing.	
31	The Member State has implemented a recent mass participation event.	Present in the NCD Progress Reports
32	The Member State has implemented a public awareness program to promote good nutrition.	Included
**Surveillance**		
1	A STEPS survey or comprehensive health examination survey is conducted every five years to assess the national capacity for surveillance.	Present in the NCD Progress Reports
2	The Member State has a population-based cancer registry.	
3	The Member State has a diabetes registry.	

4	A recent national adult risk factor survey has been conducted to evaluate the prevalence of harmful alcohol use.	
5	A recent national adult risk factor survey has been conducted to examine the prevalence of unhealthy diet.	Included (Data available only for 2021)
6	A recent national adult risk factor survey has been conducted to assess the prevalence of physical inactivity.	
7	A recent national adult risk factor survey has been conducted to evaluate the prevalence of tobacco use.	
8	A recent national adult risk factor survey has been conducted to examine the prevalence of raised blood glucose/diabetes.	
9	A recent national adult risk factor survey has been conducted to evaluate the prevalence of raised total cholesterol.	
10	A recent national adult risk factor survey has been conducted to assess the prevalence of raised blood pressure/hypertension.	
11	A recent national adult risk factor survey has been conducted to examine the prevalence of overweight and obesity.	
12	A recent national adult risk factor survey has been conducted to evaluate the prevalence of salt/sodium intake.	
13	National guidelines, protocols, and standards based on evidence exist for primary care management of major non-communicable diseases.	
14	National guidelines, protocols, and standards based on evidence exist for managing alcohol use disorders.	
15	Evidence-based national guidelines, protocols, and standards exist for cancer management.	
**Management of NCDs: Guidelines**		
1	National guidelines, protocols, and standards based on evidence exist for managing cardiovascular diseases.	
2	National guidelines, protocols, and standards based on evidence exist for managing chronic respiratory diseases.	
3	Evidence-based national guidelines, protocols, and standards exist for diabetes management.	
4	National guidelines, protocols, and standards based on evidence exist for managing overweight/obesity.	
5	National guidelines, protocols, and standards based on evidence exist for managing physical inactivity.	
6	Evidence-based national guidelines, protocols, and standards exist for managing tobacco dependence.	
**Management of NCDs: Primary and secondary prevention of cancer**		
1	There is a national program for screening cervical cancer in the country.	
2	Breast cancer screening is a part of the national screening program.	
3	The most commonly utilized screening approach in the national cervical cancer screening program.	

4	Percentage of women aged 30-49 years who undergo cervical cancer screening.	
**Management of NCDs: Procedures for managing NCDs at the upper tiers of the public healthcare system**		
1	More than half of the public sector health facilities should have provisions for acute stroke care and rehabilitation.	
2	Alteplase, a medication used for acute stroke management, should be generally available in the public health system.	
3	The public health system should have general availability of coronary bypass or stenting procedures.	
4	Diabetic retinopathy screening should be generally available in the public health system.	
5	Dialysis procedures should be generally available in the public health system.	
6	Renal replacement through transplantation should be generally available in the public health system.	
7	Retinal photocoagulation should be generally available in the public health system.	
8	The public health system should have general availability of thrombolytic therapy.	
**Management of NCDs: The provision of palliative care within the public health system**		
1	The public health system offers palliative care in community or home-based settings.	
2	Palliative care is generally available in primary health care through the public health system.	
**NCD-related tests and procedures in primary health care**		
1	Cardiovascular risk stratification should be available in at least 50% of primary health care facilities.	
2	Bone marrow transplantation should be generally available in the public health system.	
3	Blood pressure measurement should be generally available at the primary health care level.	
4	Diabetes testing by blood glucose measurement and OGTT should be generally available at the primary health care level.	
5	Diabetes testing by HbA1c should be generally available at the primary health care level.	
6	Dilated fundus examination should be generally available at the primary health care level.	
7	Foot vibration perception by tuning fork should be generally available at the primary health care level.	
8	Peak flow measurement should be generally available at the primary health care level.	
9	Spirometry should be generally available at the primary health care level.	
10	Total cholesterol measurement should be generally available at the primary health care level.	
11	Urine strips for glucose and ketone measurement should be generally available at the primary health care level.	
12	Urine testing for albumin should be generally available at the primary health care level.	
**NCD-related medicines**		
1	Public health sector offers ACE inhibitors in general.	

2	Public health sector offers Angiotensin II receptor blockers (ARBs) in general.	
3	Public health sector offers aspirin (100 mg) in general.	
4	Public health sector offers beta blockers in general.	
5	Public health sector offers bronchodilators in general.	
6	Public health sector offers CC blockers in general.	
7	Public health sector offers combination budesonide formoterol inhaler in general.	
8	Public health sector offers Fixed dose combination (lisinopril + amlodipine) in general.	
9	Public health sector offers Fixed dose combination (lisinopril + hydrochlorothiazide) in general.	
10	Public health sector offers Fixed dose combination (telmisartan + amlodipine) in general.	
11	Public health sector offers Fixed dose combination (telmisartan + hydrochlorothiazide) in general.	
12	Public health sector offers metformin in general.	
13	Public health sector offers insulin in general.	
14	Public health sector offers nicotine replacement therapy in general.	
15	Public health sector offers oral morphine in general.	
16	Public health sector offers statins in general.	
17	Public health sector offers steroid inhalers in general.	
18	Public health sector offers sulphonylurea(s) in general.	
19	Public health sector offers thiazide diuretics in general.	

Table S2. Status in the implementation of national nutrition-related policies (2017-2021)

Policy	Status	Count 2017	Percent 2017	Count 2019	Percent 2019	Count 2021	Percent 2021
Breast-milk Substitutes Marketing Code	Full	39	20,1	35	18,0	25	12,9
Breast-milk Substitutes Marketing Code	Partial	96	49,5	101	52,1	111	57,2
Child Food Marketing Restrictions	Full	68	35,1	60	30,9	73	37,6
Comprehensive Diet and Nutrition Plan	Full	152	78,4	155	79,9	162	83,5
Dietary Awareness Efforts	Full	150	77,3	71	36,6	108	55,7
Dietary Surveys	Full					92	47,4
Fat Consumption Reduction Policy	Full	68	35,1	59	30,4	55	28,4
Fat Consumption Reduction Policy	Partial			17	8,8	24	12,4
Food Taxes for NCD Reduction	Full					25	12,9
Mortality Data Availability	Full	74	38,1	78	40,2	82	42,3
Mortality Data Availability	Partial	47	24,2	40	20,6	42	21,6
Multisectoral NCD Committee	Full	71	36,6	89	45,9	89	45,9
NCD Reduction Targets	Full	94	48,5	111	57,2	109	56,2
NCD Reduction Targets	Partial	28	14,4	21	10,8	17	8,8
NCD Risk Factor Surveys	Full	37	19,1	52	26,8	36	18,6

NCD Risk Factor Surveys	Partial	135	69,6	114	58,8	125	64,4
Operational NCD Control Unit	Full	128	66,0	148	76,3	143	73,7
Operational NCD Plan	Full	98	50,5	111	57,2	107	55,2
Operational NCD Plan	Partial	22	11,3	18	9,3	13	6,7
Price Subsidies for Healthy Foods	Full					15	7,7
Salt Reduction Policy	Full	51	26,3	38	19,6	33	17,0
Salt Reduction Policy	Partial	34	17,5	46	23,7	64	33,0
SSB Tax	Full	45	23,2	74	38,1	91	46,9

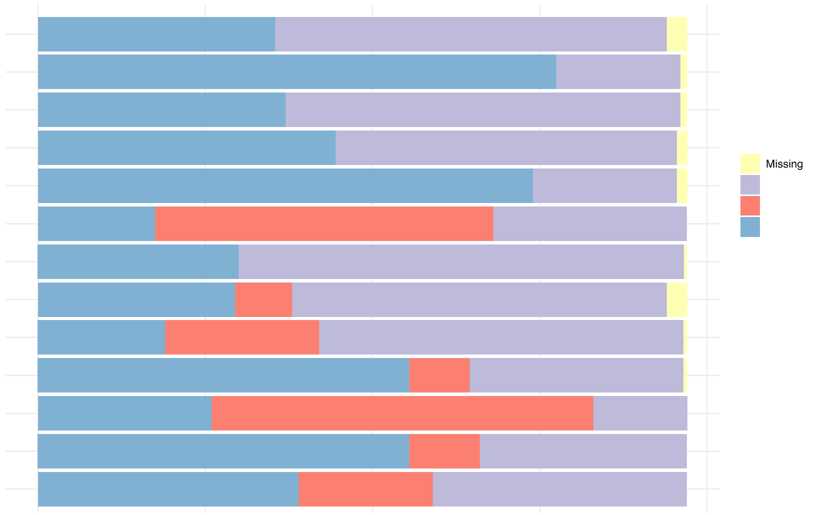

Figure S1. Implementation Status of Nutrition-Related Policies and Strategies in 2019

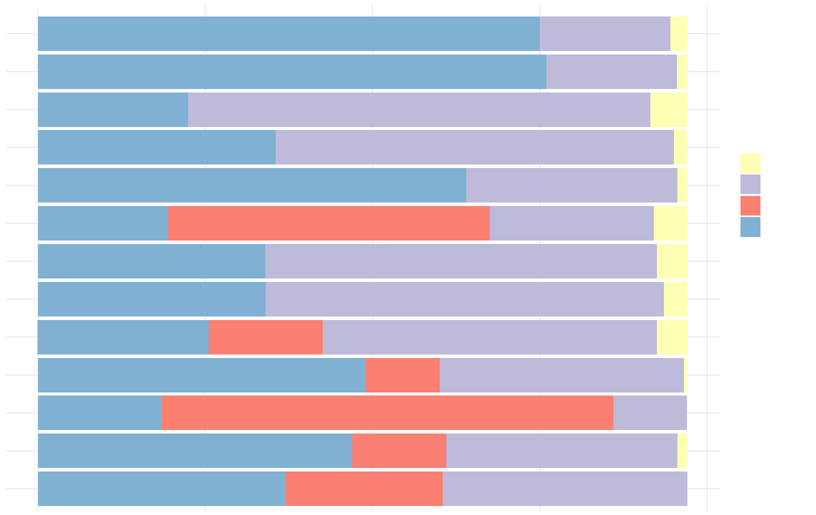

Figure S2. Implementation Status of Nutrition-Related Policies and Strategies in 2017

Table S3. Nutrition-related aggregate scores for policy implementation, ranked by aggregate score in the period 2017-2021 in each country

Country	2017	2019	2021	Δ 2017-2021	Variance
Turkey	9	11,5	12,5	3,5	3,25
Finland	10,5	12,5	12,5	2	1,33
United Arab Emirates	10,5	11	12,5	2	1,08
Chile	10	12	12	2	1,33
Iran	12	12	12	0	0
Saudi Arabia	12	10,5	12	0	0,75
Uzbekistan	8	10	11,5	3,5	3,08
Bahrain	10,5	12	11,5	1	0,58
United Kingdom	11	12	11,5	0,5	0,25
Portugal	11,5	12	11,5	0	0,08
Brazil	13	11,5	11,5	-1,5	0,75
Uruguay	8	10,5	11	3	2,58
Morocco	8,5	10	11	2,5	1,58
Tajikistan	8,5	7	11	2,5	4,08
Thailand	8,5	11	11	2,5	2,08
Brunei Darussalam	9	8	11	2	2,33
Kuwait	9,5	10	11	1,5	0,58
Russian Federation	9,5	8,5	11	1,5	1,58
Slovenia	9,5	9,5	11	1,5	0,75
Lithuania	11	11	11	0	0
Norway	11	12,5	11	0	0,75
Philippines	3,5	8,5	10,5	7	13
Tunisia	7,5	11	10,5	3	3,58
Seychelles	8	8	10,5	2,5	2,08
India	8,5	12	10,5	2	3,08
Germany	9,5	10,5	10,5	1	0,33
France	11	11,5	10,5	-0,5	0,25
Ukraine	1,5	6,5	10	8,5	18,25
Kiribati	3	7	10	7	12,33
El Salvador	7	8	10	3	2,33
Ireland	7	10,5	10	3	3,58
Poland	7,5	9,5	10	2,5	1,75

Maldives	8	8	10	2	1,33
Oman	8,5	11,5	10	1,5	2,25
Belarus	10	10	10	0	0
Bulgaria	10	10	10	0	0
Malaysia	10	9	10	0	0,33
United States of America	11	10,5	10	-1	0,25
Albania	6,5	8	9,5	3	2,25
Cook Islands	6,5	7,5	9,5	3	2,33
Belgium	7,5	10,5	9,5	2	2,33
Czech Republic	8	9,5	9,5	1,5	0,75
Kyrgyz Republic	9	8	9,5	0,5	0,58
South Africa	9	8,5	9,5	0,5	0,25
Spain	9	8,5	9,5	0,5	0,25
Italy	9,5	10,5	9,5	0	0,33
Singapore	9,5	9,5	9,5	0	0
Canada	10	10,5	9,5	-0,5	0,25
China	10	7,5	9,5	-0,5	1,75
Mongolia	10	9,5	9,5	-0,5	0,08
Qatar	10	8,5	9,5	-0,5	0,58
Costa Rica	10,5	10	9,5	-1	0,25
Estonia	10,5	10	9,5	-1	0,25
Latvia	11,5	11,5	9,5	-2	1,33
Saint Vincent and the Grenadines	4	8	9	5	7
Croatia	6	7	9	3	2,33
Peru	8	9	9	1	0,33
Panama	8,5	6,5	9	0,5	1,75
Armenia	9	8,5	9	0	0,08
Switzerland	9,5	8,5	9	-0,5	0,25
Azerbaijan	11	10	9	-2	1
Bangladesh	5	7,5	8,5	3,5	3,25
Sweden	5	6,5	8,5	3,5	3,08
Kenya	7	9	8,5	1,5	1,08
Fiji	8	6,5	8,5	0,5	1,08
Vanuatu	8	5,5	8,5	0,5	2,58
Sri Lanka	8,5	10	8,5	0	0,75

Ecuador	9	8,5	8,5	-0,5	0,08
Israel	9	8,5	8,5	-0,5	0,08
Iraq	10	7	8,5	-1,5	2,25
Greece	4	5	8	4	4,33
Iceland	6	7,5	8	2	1,08
Bosnia and Herzegovina	6,5	6	8	1,5	1,08
Benin	7,5	7,5	8	0,5	0,08
Mexico	9	9,5	8	-1	0,58
Montenegro	9	7,5	8	-1	0,58
Denmark	10	9	8	-2	1
Argentina	10,5	10,5	8	-2,5	2,08
Luxembourg	4	6,5	7,5	3,5	3,25
Kazakhstan	6,5	11,5	7,5	1	7
Palau	6,5	6,5	7,5	1	0,33
Austria	7	7,5	7,5	0,5	0,08
Serbia	7	6	7,5	0,5	0,58
Algeria	7,5	4,5	7,5	0	3
Japan	7,5	6	7,5	0	0,75
Nepal	7,5	7	7,5	0	0,08
Colombia	8	8,5	7,5	-0,5	0,25
Netherlands	8	7,5	7,5	-0,5	0,08
Mauritius	8,5	5	7,5	-1	3,25
Guatemala	9,5	9	7,5	-2	1,08
Malta	11	10,5	7,5	-3,5	3,58
Nigeria	1,5	3,5	7	5,5	7,75
Tuvalu	1,5	3	7	5,5	8,08
Nauru	5	4	7	2	2,33
Egypt	5,5	7,5	7	1,5	1,08
Slovak Republic	5,5	9	7	1,5	3,08
Cuba	6	5	7	1	1
Viet Nam	7	7	7	0	0
Moldova	10,5	11	7	-3,5	4,75
Turkmenistan	11	11	7	-4	5,33
Senegal	1	5	6,5	5,5	8,08
Rwanda	4	2	6,5	2,5	5,08
United Republic of Tanzania	5,5	7,5	6,5	1	1

Romania	6,5	7	6,5	0	0,08
Solomon Islands	6,5	5,5	6,5	0	0,33
Mozambique	7,5	5,5	6,5	-1	1
Australia	8	5,5	6,5	-1,5	1,58
Hungary	9,5	7,5	6,5	-3	2,33
Georgia	11	10,5	6,5	-4,5	6,08
Malawi	3	3	6	3	3
Venezuela	3,5	5,5	6	2,5	1,75
Samoa	4	5	6	2	1
Bahamas	4,5	6	6	1,5	0,75
Ethiopia	4,5	6,5	6	1,5	1,08
Niger	5	5,5	6	1	0,25
Suriname	5	6	6	1	0,33
Belize	5,5	5	6	0,5	0,25
Cambodia	6	5,5	6	0	0,08
Paraguay	7	6	6	-1	0,33
Tonga	7,5	7	6	-1,5	0,58
Barbados	8,5	7	6	-2,5	1,58
Bhutan	8,5	6	6	-2,5	2,08
Micronesia	4	4	5,5	1,5	0,75
Uganda	4	5	5,5	1,5	0,58
Chad	4,5	4	5,5	1	0,58
Lao PDR	4,5	3,5	5,5	1	1
Madagascar	5,5	4	5,5	0	0,75
Monaco	5,5	4	5,5	0	0,75
Guyana	7,5	6,5	5,5	-2	1
Dominican Republic	8	7	5,5	-2,5	1,58
Eswatini	8	4,5	5,5	-2,5	3,25
Jordan	9,5	5,5	5,5	-4	5,33
Korea, Rep.	11	10,5	5,5	-5,5	9,25
Pakistan	1,5	1,5	5	3,5	4,08
Togo	1,5	3,5	5	3,5	3,08
Saint Lucia	3	5,5	5	2	1,75
Myanmar	4	5	5	1	0,33
Democratic People's Republic of Korea	4,5	6	5	0,5	0,58

Côte d'Ivoire	5,5	5	5	-0,5	0,08
Trinidad and Tobago	6	5	5	-1	0,33
Mali	6,5	4	5	-1,5	1,58
Nicaragua	2	2,5	4,5	2,5	1,75
Zambia	4	3	4,5	0,5	0,58
San Marino	4,5	6	4,5	0	0,75
Jamaica	8	2,5	4,5	-3,5	7,75
Mauritania	2	4	4	2	1,33
Botswana	2,5	6	4	1,5	3,08
Sierra Leone	2,5	0	4	1,5	4,08
Eritrea	3	4,5	4	1	0,58
Burundi	3,5	4,5	4	0,5	0,25
Saint Kitts and Nevis	5,5	3	4	-1,5	1,58
New Zealand	7	3,5	4	-3	3,58
Grenada	7,5	5,5	4	-3,5	3,08
Niue	1,5	0,5	3,5	2	2,33
Namibia	2,5	3,5	3,5	1	0,33
Honduras	3,5	3	3,5	0	0,08
Lebanon	4	3	3,5	-0,5	0,25
Marshall Islands	4	2,5	3,5	-0,5	0,58
Liberia	4,5	4,5	3,5	-1	0,33
Cameroon	5,5	3,5	3,5	-2	1,33
Timor-Leste	5,5	7	3,5	-2	3,08
Afghanistan	6	4,5	3,5	-2,5	1,58
Indonesia	7	6	3,5	-3,5	3,25
Central African Republic	7,5	3,5	3,5	-4	5,33
Cabo Verde	8	5,5	3,5	-4,5	5,08
Guinea	3	1	3	0	1,33
Dominica	5,5	3	3	-2,5	2,08
Burkina Faso	6	5	3	-3	2,33
Cyprus	7	4,5	3	-4	4,08
Sao Tome and Principe	0,5	1,5	2,5	2	1
Angola	1,5	1,5	2,5	1	0,33
Gabon	2,5	4	2,5	0	0,75

Bolivia	4	3,5	2,5	-1,5	0,58
North Macedonia	5	2,5	2,5	-2,5	2,08
South Sudan	0	1	2	2	1
Democratic Republic of the Congo	0,5	3	2	1,5	1,58
Comoros	1	1	2	1	0,33
Syrian Arab Republic	1	2	2	1	0,33
Andorra	3	1,5	2	-1	0,58
Gambia	3	3	2	-1	0,33
Zimbabwe	3	2	2	-1	0,33
Congo	3,5	0	2	-1,5	3,08
Djibouti	2,5	1,5	1,5	-1	0,33
Sudan	2,5	5	1,5	-1	3,25
Papua New Guinea	4	2,5	1,5	-2,5	1,58
Ghana	5,5	2,5	1,5	-4	4,33
Lesotho	6	4,5	1,5	-4,5	5,25
Antigua and Barbuda	5	4	1	-4	4,33
Equatorial Guinea	0	0	0,5	0,5	0,08
Somalia	0	0,5	0,5	0,5	0,08
Guinea-Bissau	0,5	0,5	0,5	0	0
Libya	0,5	0	0,5	0	0,08
Haiti	1	0,5	0,5	-0,5	0,08
Yemen	1	1	0,5	-0,5	0,08
